# Poly[diaqua­bis(μ_2_-5-carb­oxy-2-propyl-1*H*-imidazole-4-carboxyl­ato-κ^3^ *N* ^3^,*O* ^4^:*O* ^5^)lead(II)]

**DOI:** 10.1107/S1600536810013735

**Published:** 2010-04-21

**Authors:** Xiang Chen, Hai-Cheng Liu

**Affiliations:** a Department of Chemistry and Chemical Engineering, Henan University of Urban Construction, Pingdingshan 467044, People’s Republic of China; b Department of Environmental and Municipal Engineering, Henan University of Urban Construction, Pingdingshan 467044, People’s Republic of China

## Abstract

In the title complex, [Pb(C_8_H_9_N_2_O_4_)_2_(H_2_O)_2_]_*n*_, the eight-coordinate Pb^II^ atom lies on a twofold rotation axis and adopts a slightly distorted square-anti­prismatic N_2_O_6_ coordination geometry. The ligand donor atoms are the tertiary N atoms of the imidazole rings and the carboxyl­ate O atoms of two chelating 5-carb­oxy-2-propyl-1*H*-imidazole-4-carboxyl­ate ligands, the carb­oxy O atoms of two additional imidazole ligands and two water O atoms. The carb­oxy O and the *N*,*O*-chelate systems also link adjacent Pb^II^ atoms, forming a two-dimensional layer structure, with four individual Pb^II^ atoms located at the corners of a square. These layers are further inter­connected by an extensive array of O—H⋯O and N—H⋯O hydrogen bonds into a three-dimensional network.

## Related literature

For the properties and uses of imidazole­dicarboxyl­ate complexes, see: Cao *et al.* (2002 ▶); Rajendiran *et al.* (2003 ▶).
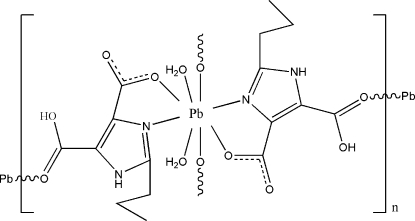


## Experimental

### 

#### Crystal data

[Pb(C_8_H_9_N_2_O_4_)_2_(H_2_O)_2_]
*M* *_r_* = 637.57
Monoclinic, 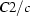

*a* = 13.1201 (15) Å
*b* = 13.2929 (16) Å
*c* = 11.5910 (13) Å
β = 98.531 (2)°
*V* = 1999.2 (4) Å^3^
*Z* = 4
Mo *K*α radiation
μ = 8.50 mm^−1^
*T* = 293 K
0.45 × 0.17 × 0.13 mm

#### Data collection

Bruker SMART 1000 CCD area-detector diffractometer
Absorption correction: multi-scan (*SADABS*; Bruker, 2007 ▶) *T*_min_ = 0.114, *T*_max_ = 0.404
4884 measured reflections
1751 independent reflections
1640 reflections with *I* > 2σ(*I*)
*R*_int_ = 0.048

#### Refinement

*R*[*F* ^2^ > 2σ(*F* ^2^)] = 0.019
*wR*(*F* ^2^) = 0.039
*S* = 1.02
1751 reflections
143 parameters
H-atom parameters constrained
Δρ_max_ = 0.82 e Å^−3^
Δρ_min_ = −0.69 e Å^−3^

### 

Data collection: *SMART* (Bruker, 2007 ▶); cell refinement: *SAINT* (Bruker, 2007 ▶); data reduction: *SAINT*; program(s) used to solve structure: *SHELXS97* (Sheldrick, 2008 ▶); program(s) used to refine structure: *SHELXL97* (Sheldrick, 2008 ▶); molecular graphics: *SHELXTL* (Sheldrick, 2008 ▶) and *DIAMOND* (Brandenburg, 1999 ▶); software used to prepare material for publication: *SHELXTL*.

## Supplementary Material

Crystal structure: contains datablocks I, global. DOI: 10.1107/S1600536810013735/sj2771sup1.cif

Structure factors: contains datablocks I. DOI: 10.1107/S1600536810013735/sj2771Isup2.hkl

Additional supplementary materials:  crystallographic information; 3D view; checkCIF report

## Figures and Tables

**Table 1:** Hydrogen-bond geometry (Å, °)

*D*—H⋯*A*	*D*—H	H⋯*A*	*D*⋯*A*	*D*—H⋯*A*
O5—H5*D*⋯O4^i^	0.85	2.58	3.182 (4)	129
O5—H5*D*⋯O3^i^	0.85	2.16	3.004 (3)	176
O5—H5*C*⋯O1^ii^	0.85	2.19	3.035 (3)	176
O3—H3⋯O2	0.82	1.64	2.459 (3)	178
N2—H2⋯O1^iii^	0.86	2.05	2.909 (4)	172

Symmetry codes: (i) 
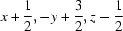
; (ii) 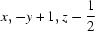; (iii) 
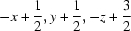
.

**Fig. 1.**
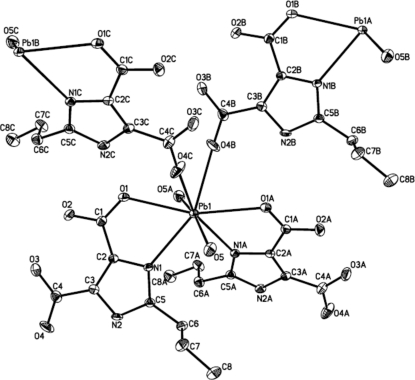
The structure of the title compound, showing the atomic numbering scheme. Non-H atoms are shown with 30% probability displacement ellipsoids(H atoms are omitted for clarity). [Symmetry codes: (i) 1-x, y, 1.5-z; (ii) -0.5+x, 0.5+y, z; (iii) 0.5+x, 0.5+y, z; (iv) 0.5+x, -0.5+y, z; (v) 0.5-x, -0.5+y, 1.5-z]

**Fig. 2.**
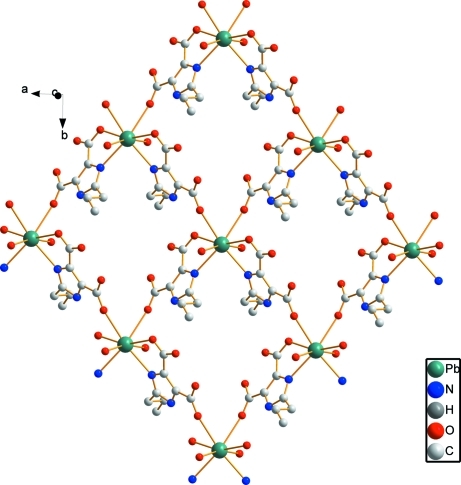
A two-dimensional layer constructed of Pb4 squares

**Fig. 3.**
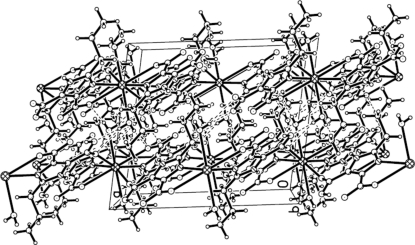
Crystal packing of the title compound

## supplementary crystallographic information

### Comment

It is well known that 4,5-imidazoledicarboxylic acid, a rigid N-heterocyclic dicarboxylic acid has great potential as a ligand in coordination complexes and for hydrogen bond formation. Imidazoledicarboxylate complexes have been found to exhibit useful properties, such as magnetism and porosity (Cao *et al.*, 2002; Rajendiran *et al.*, 2003). We have therefore reacted the 2-propyl-1*H*-imidazole-4,5 dicarboxylic acid ligand with Pb(NO_3_)_2_ under hydrothermal conditions to obtain a new Pb^II^ complex and its structure is reported here.

In the title complex, [Pb(C_8_H_9_N_2_O_4_)_2_(H_2_O)_2_]_n_, the eight coordinate Pb^II^ atom lies on a two-fold rotation axis and adopts a slightly distorted square-antiprismatic N_2_O_6_ coordination geometry. The ligand donor atoms are the N1 atoms of the imidazole rings and the carboxylate O1 atoms of two chelating 5-carboxy-2-propyl-1*H*-imidazole-4- carboxylato ligands, the O4 carboxy oxygen atoms of two additional imidazole ligands and two O5 water molecules. The O1 and N1 atoms of the chelate systems also link adjacent Pb^II^ centres forming a two-dimensional layer structure, with four individual Pb^II^ atoms located at the corners of a square. These layers are further interconnected by an extensive array of O—H···O and N—H···O hydrogen bonds into a three-dimensional network.

### Experimental

A mixture of Pb(NO_3_)_2_ (0.5 mmol, 0.07 g) and 2-propyl-1*H*-imidazole-4,5-dicarboxylic acid(0.5 mmol, 0.99 g) in 15 ml of H_2_O solution was sealed in an autoclave equipped with a Teflon liner (25 ml) and then heated at 373k for 3 days. Crystals of the title compound were obtained by slow evaporation of the solvent at room temperature.

### Refinement

Carbon and nitrogen bound H atoms were placed at calculated positions and were treated as riding on the parent C or N atoms with C—H = 0.93 Å, N—H = 0.86 Å, and with *U*_iso_(H) = 1.2 *U*_eq_(C, N). H atoms of the water molecule were located in a difference map and were allowed to ride on the parent atom, with *U*_iso_(H)=1.2 *U*_eq_.

### Crystal data

**Table:**

[Pb(C_8_H_9_N_2_O_4_)_2_(H_2_O)_2_]	*F*(000) = 1232
*M**_r_* = 637.57	*D*_x_ = 2.118 Mg m^−^^3^
Monoclinic, *C*2/*c*	Mo *K*α radiation, λ = 0.71073 Å
Hall symbol: -C 2yc	Cell parameters from 2051 reflections
*a* = 13.1201 (15) Å	θ = 2.5–23.9°
*b* = 13.2929 (16) Å	µ = 8.50 mm^−^^1^
*c* = 11.5910 (13) Å	*T* = 293 K
β = 98.531 (2)°	Block, colorless
*V* = 1999.2 (4) Å^3^	0.45 × 0.17 × 0.13 mm
*Z* = 4	

### Data collection

**Table:**

Bruker SMART 1000 CCD area-detector diffractometer	1751 independent reflections
Radiation source: fine-focus sealed tube	1640 reflections with *I* > 2σ(*I*)
graphite	*R*_int_ = 0.048
φ and ω scans	θ_max_ = 25.0°, θ_min_ = 2.2°
Absorption correction: multi-scan (*SADABS*; Bruker, 2007)	*h* = −13→15
*T*_min_ = 0.114, *T*_max_ = 0.404	*k* = −15→15
4884 measured reflections	*l* = −10→13

### Refinement

**Table:**

Refinement on *F*^2^	Secondary atom site location: difference Fourier map
Least-squares matrix: full	Hydrogen site location: inferred from neighbouring sites
*R*[*F*^2^ > 2σ(*F*^2^)] = 0.019	H-atom parameters constrained
*wR*(*F*^2^) = 0.039	*w* = 1/[σ^2^(*F*_o_^2^) + 0.170*P*] where *P* = (*F*_o_^2^ + 2*F*_c_^2^)/3
*S* = 1.02	(Δ/σ)_max_ = 0.002
1751 reflections	Δρ_max_ = 0.82 e Å^−^^3^
143 parameters	Δρ_min_ = −0.69 e Å^−^^3^
0 restraints	Extinction correction: *SHELXL97* (Sheldrick, 2008), Fc^*^=kFc[1+0.001xFc^2^λ^3^/sin(2θ)]^-1/4^
Primary atom site location: structure-invariant direct methods	Extinction coefficient: 0.00501 (14)

### Special details

**Table:**

Geometry. All esds (except the esd in the dihedral angle between two l.s. planes) are estimated using the full covariance matrix. The cell esds are taken into account individually in the estimation of esds in distances, angles and torsion angles; correlations between esds in cell parameters are only used when they are defined by crystal symmetry. An approximate (isotropic) treatment of cell esds is used for estimating esds involving l.s. planes.
Refinement. Refinement of *F*^2^ against ALL reflections. The weighted *R*-factor wR and goodness of fit *S* are based on *F*^2^, conventional *R*-factors *R* are based on *F*, with *F* set to zero for negative *F*^2^. The threshold expression of *F*^2^ > σ(*F*^2^) is used only for calculating *R*-factors(gt) etc. and is not relevant to the choice of reflections for refinement. *R*-factors based on *F*^2^ are statistically about twice as large as those based on *F*, and *R*- factors based on ALL data will be even larger.

### Fractional atomic coordinates and isotropic or equivalent isotropic displacement parameters (Å^2^)

**Table:**

	*x*	*y*	*z*	*U*_iso_*/*U*_eq_	
Pb1	0.5000	0.585420 (12)	0.7500	0.02187 (10)	
N1	0.36101 (17)	0.72953 (19)	0.7416 (2)	0.0206 (6)	
N2	0.26176 (19)	0.8636 (2)	0.7255 (2)	0.0245 (6)	
H2	0.2380	0.9221	0.7040	0.029*	
O1	0.33533 (16)	0.55121 (18)	0.8607 (2)	0.0288 (6)	
O2	0.20043 (17)	0.61233 (19)	0.9317 (2)	0.0346 (6)	
O3	0.09662 (17)	0.7669 (2)	0.9230 (2)	0.0400 (7)	
H3	0.1304	0.7149	0.9245	0.060*	
O4	0.0980 (2)	0.9168 (2)	0.8428 (3)	0.0521 (9)	
O5	0.43915 (18)	0.6096 (2)	0.5232 (2)	0.0374 (6)	
H5C	0.4120	0.5656	0.4750	0.045*	
H5D	0.4830	0.6427	0.4915	0.045*	
C1	0.2736 (2)	0.6209 (3)	0.8716 (3)	0.0240 (7)	
C2	0.2842 (2)	0.7169 (2)	0.8095 (3)	0.0204 (7)	
C3	0.2221 (2)	0.8003 (3)	0.7997 (3)	0.0228 (8)	
C4	0.1323 (3)	0.8316 (3)	0.8572 (3)	0.0314 (9)	
C5	0.3450 (2)	0.8188 (2)	0.6912 (3)	0.0230 (8)	
C6	0.4039 (2)	0.8661 (3)	0.6046 (3)	0.0285 (8)	
H6A	0.4688	0.8309	0.6062	0.034*	
H6B	0.4193	0.9353	0.6274	0.034*	
C7	0.3461 (3)	0.8646 (3)	0.4806 (3)	0.0355 (9)	
H7A	0.3380	0.7954	0.4541	0.043*	
H7B	0.2779	0.8928	0.4803	0.043*	
C8	0.4016 (3)	0.9236 (3)	0.3967 (4)	0.0408 (10)	
H8A	0.4129	0.9913	0.4246	0.061*	
H8B	0.3604	0.9245	0.3210	0.061*	
H8C	0.4667	0.8923	0.3914	0.061*	

### Atomic displacement parameters (Å^2^)

**Table:**

	*U*^11^	*U*^22^	*U*^33^	*U*^12^	*U*^13^	*U*^23^
Pb1	0.02470 (12)	0.01520 (13)	0.02690 (14)	0.000	0.00777 (7)	0.000
N1	0.0251 (13)	0.0146 (16)	0.0229 (15)	0.0002 (11)	0.0059 (11)	0.0026 (13)
N2	0.0337 (15)	0.0126 (15)	0.0273 (16)	0.0049 (12)	0.0047 (12)	0.0059 (14)
O1	0.0343 (13)	0.0162 (13)	0.0375 (15)	0.0015 (10)	0.0110 (10)	0.0029 (12)
O2	0.0376 (14)	0.0306 (14)	0.0396 (16)	−0.0018 (11)	0.0191 (11)	0.0072 (13)
O3	0.0354 (13)	0.0414 (18)	0.0474 (17)	0.0086 (12)	0.0203 (11)	0.0008 (15)
O4	0.0632 (18)	0.046 (2)	0.051 (2)	0.0373 (14)	0.0236 (15)	0.0114 (15)
O5	0.0407 (14)	0.0395 (16)	0.0338 (16)	−0.0070 (12)	0.0115 (11)	−0.0025 (13)
C1	0.0266 (17)	0.0222 (19)	0.0236 (19)	−0.0044 (15)	0.0049 (14)	−0.0031 (17)
C2	0.0235 (15)	0.0163 (18)	0.0212 (18)	−0.0013 (13)	0.0026 (13)	−0.0018 (15)
C3	0.0264 (16)	0.022 (2)	0.0198 (18)	0.0019 (14)	0.0022 (13)	−0.0012 (16)
C4	0.0335 (19)	0.035 (2)	0.027 (2)	0.0074 (17)	0.0083 (15)	−0.0017 (19)
C5	0.0244 (16)	0.0178 (19)	0.0266 (19)	−0.0001 (14)	0.0030 (13)	0.0008 (16)
C6	0.0286 (18)	0.025 (2)	0.033 (2)	−0.0009 (15)	0.0050 (14)	0.0066 (18)
C7	0.0365 (19)	0.037 (2)	0.033 (2)	−0.0111 (17)	0.0026 (15)	0.003 (2)
C8	0.049 (2)	0.042 (3)	0.031 (2)	−0.0065 (18)	0.0058 (18)	0.0088 (19)

### Geometric parameters (Å, °)

**Table:**

Pb1—N1	2.637 (2)	O4—C4	1.221 (4)
Pb1—N1^i^	2.637 (2)	O4—Pb1^iv^	2.725 (3)
Pb1—O5^i^	2.651 (3)	O5—H5C	0.8500
Pb1—O5	2.652 (3)	O5—H5D	0.8500
Pb1—O1	2.710 (2)	C1—C2	1.481 (5)
Pb1—O1^i^	2.710 (2)	C2—C3	1.370 (4)
Pb1—O4^ii^	2.725 (3)	C3—C4	1.496 (5)
Pb1—O4^iii^	2.725 (3)	C5—C6	1.494 (5)
N1—C5	1.326 (4)	C6—C7	1.522 (4)
N1—C2	1.377 (4)	C6—H6A	0.9700
N2—C5	1.354 (4)	C6—H6B	0.9700
N2—C3	1.361 (5)	C7—C8	1.517 (5)
N2—H2	0.8600	C7—H7A	0.9700
O1—C1	1.249 (4)	C7—H7B	0.9700
O2—C1	1.273 (4)	C8—H8A	0.9600
O3—C4	1.284 (4)	C8—H8B	0.9600
O3—H3	0.8200	C8—H8C	0.9600
			
N1—Pb1—N1^i^	86.81 (11)	Pb1—O5—H5C	127.1
N1—Pb1—O5^i^	93.06 (8)	Pb1—O5—H5D	111.6
N1^i^—Pb1—O5^i^	76.75 (8)	H5C—O5—H5D	108.5
N1—Pb1—O5	76.75 (8)	O1—C1—O2	122.8 (3)
N1^i^—Pb1—O5	93.06 (8)	O1—C1—C2	118.7 (3)
O5^i^—Pb1—O5	166.09 (11)	O2—C1—C2	118.4 (3)
N1—Pb1—O1	62.85 (7)	C3—C2—N1	109.3 (3)
N1^i^—Pb1—O1	134.44 (7)	C3—C2—C1	129.8 (3)
O5^i^—Pb1—O1	72.23 (7)	N1—C2—C1	120.8 (3)
O5—Pb1—O1	110.24 (7)	N2—C3—C2	106.0 (3)
N1—Pb1—O1^i^	134.44 (7)	N2—C3—C4	120.6 (3)
N1^i^—Pb1—O1^i^	62.85 (7)	C2—C3—C4	133.3 (3)
O5^i^—Pb1—O1^i^	110.23 (7)	O4—C4—O3	122.8 (3)
O5—Pb1—O1^i^	72.23 (7)	O4—C4—C3	119.8 (4)
O1—Pb1—O1^i^	160.68 (10)	O3—C4—C3	117.3 (3)
N1—Pb1—O4^ii^	153.57 (9)	N1—C5—N2	110.3 (3)
N1^i^—Pb1—O4^ii^	107.45 (9)	N1—C5—C6	127.6 (3)
O5^i^—Pb1—O4^ii^	69.71 (9)	N2—C5—C6	122.1 (3)
O5—Pb1—O4^ii^	123.10 (9)	C5—C6—C7	113.2 (3)
O1—Pb1—O4^ii^	92.16 (8)	C5—C6—H6A	108.9
O1^i^—Pb1—O4^ii^	71.71 (8)	C7—C6—H6A	108.9
N1—Pb1—O4^iii^	107.46 (9)	C5—C6—H6B	108.9
N1^i^—Pb1—O4^iii^	153.56 (9)	C7—C6—H6B	108.9
O5^i^—Pb1—O4^iii^	123.10 (9)	H6A—C6—H6B	107.8
O5—Pb1—O4^iii^	69.70 (9)	C8—C7—C6	112.3 (3)
O1—Pb1—O4^iii^	71.72 (8)	C8—C7—H7A	109.1
O1^i^—Pb1—O4^iii^	92.16 (8)	C6—C7—H7A	109.1
O4^ii^—Pb1—O4^iii^	69.29 (14)	C8—C7—H7B	109.1
C5—N1—C2	106.2 (3)	C6—C7—H7B	109.1
C5—N1—Pb1	136.9 (2)	H7A—C7—H7B	107.9
C2—N1—Pb1	116.79 (19)	C7—C8—H8A	109.5
C5—N2—C3	108.3 (3)	C7—C8—H8B	109.5
C5—N2—H2	125.9	H8A—C8—H8B	109.5
C3—N2—H2	125.9	C7—C8—H8C	109.5
C1—O1—Pb1	119.8 (2)	H8A—C8—H8C	109.5
C4—O3—H3	109.5	H8B—C8—H8C	109.5
C4—O4—Pb1^iv^	163.7 (3)		
			
N1^i^—Pb1—N1—C5	39.1 (3)	Pb1—N1—C2—C1	−7.6 (3)
O5^i^—Pb1—N1—C5	115.6 (3)	O1—C1—C2—C3	174.0 (3)
O5—Pb1—N1—C5	−54.8 (3)	O2—C1—C2—C3	−4.4 (5)
O1—Pb1—N1—C5	−176.2 (3)	O1—C1—C2—N1	−0.5 (4)
O1^i^—Pb1—N1—C5	−6.9 (3)	O2—C1—C2—N1	−178.9 (3)
O4^ii^—Pb1—N1—C5	163.3 (3)	C5—N2—C3—C2	0.4 (4)
O4^iii^—Pb1—N1—C5	−118.2 (3)	C5—N2—C3—C4	176.8 (3)
N1^i^—Pb1—N1—C2	−137.1 (2)	N1—C2—C3—N2	0.0 (3)
O5^i^—Pb1—N1—C2	−60.6 (2)	C1—C2—C3—N2	−175.0 (3)
O5—Pb1—N1—C2	129.0 (2)	N1—C2—C3—C4	−175.8 (3)
O1—Pb1—N1—C2	7.63 (19)	C1—C2—C3—C4	9.3 (6)
O1^i^—Pb1—N1—C2	176.89 (18)	Pb1^iv^—O4—C4—O3	−108.0 (9)
O4^ii^—Pb1—N1—C2	−12.9 (3)	Pb1^iv^—O4—C4—C3	74.0 (10)
O4^iii^—Pb1—N1—C2	65.6 (2)	N2—C3—C4—O4	−4.3 (5)
N1—Pb1—O1—C1	−8.6 (2)	C2—C3—C4—O4	171.0 (4)
N1^i^—Pb1—O1—C1	45.2 (3)	N2—C3—C4—O3	177.7 (3)
O5^i^—Pb1—O1—C1	94.6 (2)	C2—C3—C4—O3	−7.1 (6)
O5—Pb1—O1—C1	−70.9 (2)	C2—N1—C5—N2	0.7 (3)
O1^i^—Pb1—O1—C1	−164.9 (2)	Pb1—N1—C5—N2	−175.8 (2)
O4^ii^—Pb1—O1—C1	162.5 (2)	C2—N1—C5—C6	−176.9 (3)
O4^iii^—Pb1—O1—C1	−130.2 (2)	Pb1—N1—C5—C6	6.7 (5)
Pb1—O1—C1—O2	−173.4 (2)	C3—N2—C5—N1	−0.7 (4)
Pb1—O1—C1—C2	8.3 (4)	C3—N2—C5—C6	177.0 (3)
C5—N1—C2—C3	−0.4 (3)	N1—C5—C6—C7	105.0 (4)
Pb1—N1—C2—C3	176.90 (19)	N2—C5—C6—C7	−72.3 (4)
C5—N1—C2—C1	175.1 (3)	C5—C6—C7—C8	173.3 (3)

Symmetry codes: (i) −*x*+1, *y*, −*z*+3/2; (ii) *x*+1/2, *y*−1/2, *z*; (iii) −*x*+1/2, *y*−1/2, −*z*+3/2; (iv) *x*−1/2, *y*+1/2, *z*.

### Hydrogen-bond geometry (Å, °)

**Table:**

*D*—H···*A*	*D*—H	H···*A*	*D*···*A*	*D*—H···*A*
O5—H5D···O4^v^	0.85	2.58	3.182 (4)	129
O5—H5D···O3^v^	0.85	2.16	3.004 (3)	176
O5—H5C···O1^vi^	0.85	2.19	3.035 (3)	176
O3—H3···O2	0.82	1.64	2.459 (3)	178
N2—H2···O1^vii^	0.86	2.05	2.909 (4)	172

Symmetry codes: (v) *x*+1/2, −*y*+3/2, *z*−1/2; (vi) *x*, −*y*+1, *z*−1/2; (vii) −*x*+1/2, *y*+1/2, −*z*+3/2.
